# A Long‐Period Grating Formed by Residual Stress on Thin‐Film Lithium Niobate With Efficient Electro‐Optic and Thermo‐Optic Tuning

**DOI:** 10.1002/nap2.70118

**Published:** 2026-05-05

**Authors:** Jiayao Deng, Hao Yao, Yuzhe Sun, Hao Zhang, Yinglu Zhang, Jieyun Wu, Kaixin Chen

**Affiliations:** ^1^ School of Optoelectronic Science and Engineering University of Electronic Science and Technology of China Chengdu China

**Keywords:** long‐period waveguide grating, reconfigurable photonic devices, stress‐induced refractive index modulation, thermo‐optic tuning, thin‐film lithium niobate

## Abstract

We propose and demonstrate a long‐period waveguide grating with tunable resonant wavelength and contrast based on hybrid polymer and thin‐film lithium niobate platform. The grating is formed in the polymer cladding through stress‐induced refractive index modulation, achieved by a periodic metal structure on the cladding. This metal structure also acts as a heater for thermo‐optic (TO) tuning and an electrode for electro‐optic (EO) tuning. Thanks to the bidirectional index change provided by the EO effect, the device allows either red or blue shift of the resonant wavelength and control of the resonant contrast. The fabricated device exhibits a resonant contrast of 12.38 (19.65) dB at 1553.43 (1542.68) nm for the *x* (*z*) polarization, with EO tuning efficiencies of 20.2 (25.1) pm/V. Further annealing experiments reveal that its operation can switch from a stress‐dominated to a more linear, EO‐dominated regime. Consequently, applying +60 V increases the contrast from 24.18 to 31.86 dB, whereas −60 V completely suppresses the resonance; meanwhile, the EO tuning efficiency rises to 28.5 pm/V. Additionally, the grating achieves a maximum TO tuning efficiency of 18.6 pm/mW. Our proposed grating can be employed to implement tunable photonic devices in optical communications and information processing systems.

## Graphical abstract

Three‐dimensional schematic (a) of the proposed long‐period grating (LPG) on thin‐film lithium niobate (TFLN) and its cross‐sectional view (b) showing a TFLN core with a BCB cladding and a periodic metal structure. Residual stress generated at the metal–BCB interface creates the LPG. The metal structure also enables electro‐optic and thermo‐optic tuning of resonance wavelength and contrast of the LPG.

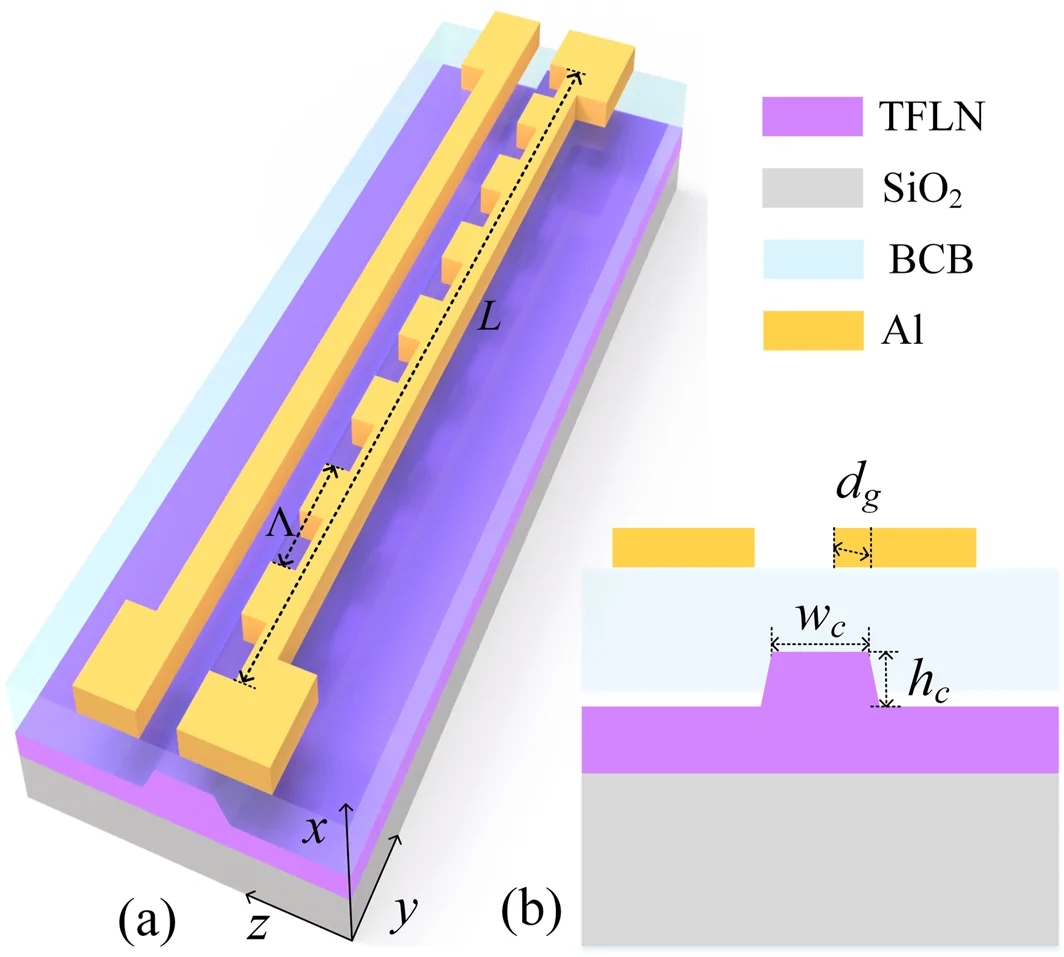

## 1 Introduction

Dynamically tunable gratings enable flexible control over the wavelength, mode, intensity, and path of light, which makes them key and versatile components for constructing tunable, reconfigurable, and even programmable photonic devices used in optical communication and optical information processing systems. These devices include tunable or reconfigurable filters [1, 2, 3, 4, 5, 6, 7, 8, 9, 10, 11, 12], mode switches [13], tunable mode filters [14], reconfigurable mode (de)multiplexers [15], modulator [16, 17, 18, 19, 20, 21, 22], tunable add–drop multiplexer [23], tunable laser [24, 25], tunable spectral broadening device [26], programmable photonic circuits [27, 28], and so on. Currently, there are two primary approaches for realizing dynamic tuning of gratings. The first involves creating permanent gratings through periodic refractive index modulation introduced by techniques such as etching or proton exchange, followed by dynamic tuning of the grating transmission spectrum using electro‐optic (EO) [1, 2, 3, 4, 5, 6], thermo‐optic (TO) [7, 8, 9, 10, 11, 12, 26, 27], or piezoelectric [25] effects. These tuning mechanisms offer different response times ranging from nanoseconds to milliseconds, among which EO tuning is the optimal choice for high‐speed operation. The advantage of this approach is that it enables the realization of tunable waveguide gratings on various material platforms. However, the invasive fabrication processes used to form these permanent gratings not only compromise the structural integrity of the waveguide, leading to significant scattering losses but also result in a fixed refractive index modulation. Consequently, whether the TO or EO tuning is employed, the tuning is primarily limited to the resonant wavelength of the grating, with low efficiency in tuning the contrast.

To address this issue, researchers have proposed stress‐induced [23, 29], TO induced [15, 30], and EO induced [13, 31, 32, 33] long‐period waveguide gratings (LPWGs). In 2006, Chiang et al. experimentally demonstrated a stress‐induced LPWG by patterning a periodic metal structure on a polymer waveguide to create a periodic refractive index modulation. This metal structure was also used as an electrode heater to achieve simultaneous thermal tuning of both the resonant wavelength and the contrast in the transmission spectrum of the grating. Regarding TO induced gratings, in 2005 [30], Kwon and Shin demonstrated a TO induced LPWG by using a periodic metallic electrode heater fabricated above a polymer waveguide to create a thermally induced periodic refractive index modulation. However, their fabricated grating required a quite large driving power of up to 600 mW to achieve a contrast greater than 10 dB. Subsequently, in 2018 [15], Zhao et al. experimentally demonstrated a similar TO induced LPWG with significantly reduced driving power. Their device achieved a high mode selectivity of 23 dB with a driving power of only 198 mW. As for EO induced gratings, in 2008 [31], Jin et al. first experimentally demonstrated an EO LPWG based on a proton‐exchanged lithium niobate (LN) waveguide through a periodic electrode to realize periodic refractive index modulation, although it required a driving voltage as high as 97 V to achieve a contrast of 27 dB. Later, they reduced the driving voltage to 29 V by employing graphene electrodes with low absorption loss [33]. Overall, compared to the first method, the second approach does not rely on invasive processes to form the gratings, thereby preserving the structural integrity of the waveguide. The grating fabrication process is relatively simple. More importantly, these induced gratings allow the strength of the grating, and hence its resonant wavelength and contrast, to be controlled via external driving, making them highly advantageous for realizing various tunable and reconfigurable devices.

In this paper, we propose and experimentally demonstrate a stress‐induced LPWG with tunable resonant wavelength and contrast, based on a hybrid polymer and thin‐film lithium niobate (TFLN) platform. The device is composed of a TFLN ridge waveguide core with a benzocyclobutene (BCB) upper cladding, along with both straight and periodic metal structures patterned on the BCB layer. Theoretical analysis indicates that the residual stress, generated at the interface between the periodic metal structure and the BCB during cooling from process temperature, can induce substantial periodic refractive index modulation in the BCB layer, thereby forming a LPWG without the need for etching. We theoretically investigated the optical characteristics of this stress‐induced grating. The results show that a contrast exceeding 25 dB can be achieved. It is particularly noteworthy that in this work, the same metal structure that induces the grating also serves as both a TO tuning electrode heater and an EO modulation electrode. When the EO tuning is employed, an EO grating with a period nearly identical to that of the stress‐induced grating is formed. Consequently, the device supports not only red or blue shift tuning of the resonant wavelength but also active control of the resonant contrast, enabling it to be enhanced, suppressed, or even completely erased. This is because the EO effect can either increase or decrease the refractive index of LN, depending on the direction of the applied electric field. Our carefully fabricated device exhibits a resonant contrast of 12.38 dB at 1553.43 nm for the *x*‐polarization and 19.65 dB at 1542.68 nm for the *z*‐polarization, with EO tuning efficiencies of 20.2 and 25.1 pm/V, respectively. Further annealing experiments revealed that annealing reduces the initial strength of the stress‐induced grating, thereby switching its operation from a stress‐dominated to a more linear EO‐dominated regime. As a result, the transmission spectrum contrast increases from 24.18 to 31.86 dB under a positive EO tuning voltage of +60 V, while a negative voltage of −60 V completely suppresses the resonance. After annealing, the EO tuning efficiency increases to 28.5 pm/V. Additionally, the grating achieves a maximum TO tuning efficiency of 18.6 pm/mW. These characteristics indicate that the proposed grating can be employed to implement tunable or reconfigurable photonic devices in optical communications and information processing systems.

## 2 Device Configuration and Principle

The proposed stress‐induced LPWG is shown schematically in Figure 1a. The device is fabricated on a commercial X‐cut TFLN platform, comprising a 4.7‐μm thick buried silicon dioxide (SiO_2_) layer and a 600‐nm thick LN film on top. Figure 1b presents the cross‐sectional view of the device. The core is a rib waveguide defined in the LN film, with a width of *w*_c_ and an etch depth of *h*_c_. A layer of BCB film serves as the upper cladding. BCB is chosen because it is a polymer material commonly used in our laboratory with a well‐established processing, and it offers a large thermal expansion coefficient (CTE) mismatch with aluminum (Al; 52 × 10^−6^/K for BCB vs. 23 × 10^−6^/K for Al). Both straight and periodic Al patterns are deposited directly onto the BCB cladding. These metal structures serve two functions: first, to enable the implementation of a stress‐induced LPWG, and second, to provide a means for tuning this grating. The first function is implemented through the thermal mismatch between this periodic metal structure and the BCB thin film. Upon cooling from the process temperature, this mismatch generates a periodic residual stress, which in turn creates a corresponding periodic modulation in the refractive index of the BCB. The second function is realized by the specific design in which the straight and periodic Al patterns are separated by a gap that varies periodically with an appropriate profile. When a voltage is applied across the two electrodes, they act as a pair of EO modulation electrodes for the EO tuning. Alternatively, applying a current solely through the periodic Al structure allows it to function as a micro‐heater for the TO tuning. The periodically varying gap between the electrodes has a minimum spacing of 2.8 μm and a maximum spacing of 6 μm. The refractive indices of the TFLN core, BCB cladding, and the buried oxide layer are *n*_c_, *n*_cl_, and *n*_s_, respectively, and at 1550 nm wavelength, *n*_c_ = 2.148 for the *z*‐polarization and 2.190 for the *x*‐polarization while *n*_cl_ = 1.538 and *n*_s_ = 1.445.

**FIGURE 1:**
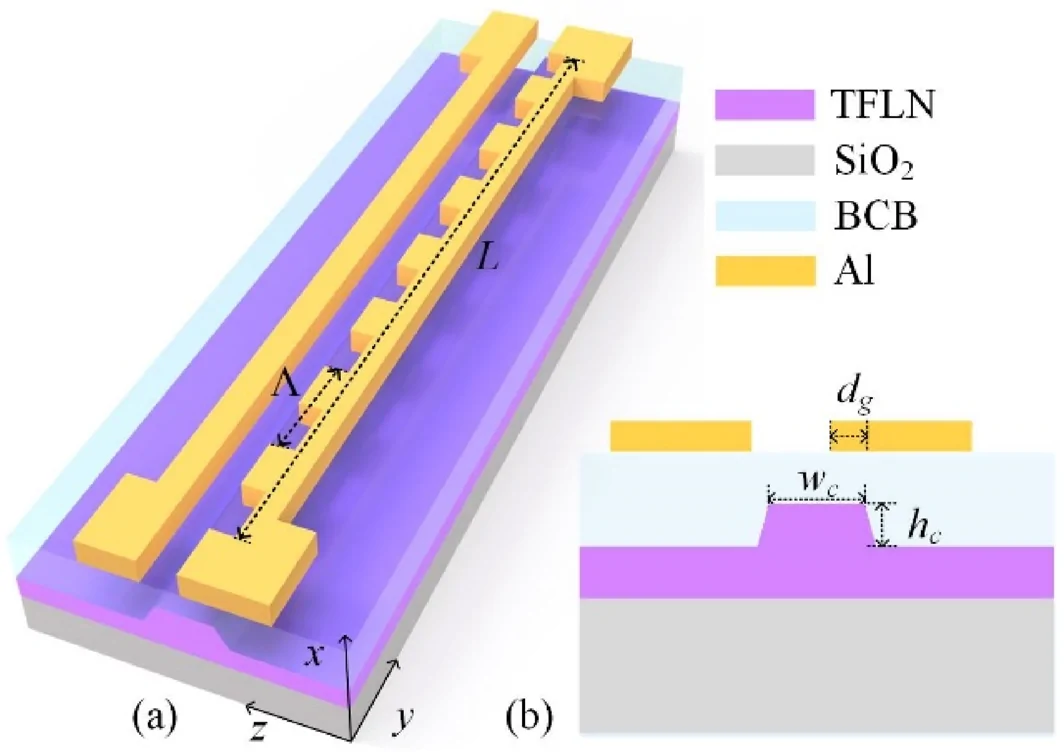
Schematic diagrams showing (a) 3D view and (b) cross‐sectional view of the device.

In this work, the grating is designed to implement the coupling between the fundamental mode and the first‐order mode in the TFLN rib waveguides. To leverage the maximum EO coefficient *γ*_33_ of LN for resonant wavelength tuning, the LPWG is optimized only for the *z*‐polarized light at 1550 nm resonant wavelength. It should be noted that the device remains functional for the *x*‐polarized light, however, its resonant wavelength for this polarization will deviate from 1550 nm. With the waveguide width *w*_c_ set to 2.8 μm and the etch depth *h*_c_ set to 280 nm, the effective refractive indices of the *z*‐polarized modes at 1550 nm, calculated with Rsoft MODE Solutions, are *N*_eff1_ = 1.9594 (fundamental mode) and *N*_eff2_ = 1.9198 (first‐order mode). Further, according to the grating phase‐matching condition [5],

(1) $$\Lambda =\lambda /\left(N_{\text{eff}1}-N_{\text{eff}2}\right),$$

the grating period Λ at the 1550 nm resonant wavelength is calculated to be 39.13 μm. As mentioned above, this grating is formed not by conventional etching processes but through stress‐induced refractive index modulation. To investigate this modulation and thus determine the grating length, we first employed COMSOL Multiphysics to calculate the strain in the BCB film. This strain originates from the significant thermal expansion coefficient mismatch between the patterned Al and the underlying BCB. Assuming a maximum temperature change of 300°C at the BCB film during Al deposition via thermal evaporation, the calculated strain distribution reveals an average tensile strain Δ*ε* of approximately −2.6 × 10^−2^ in the BCB cladding near the TFLN ridge waveguide, as shown in Figure 2.

**FIGURE 2:**
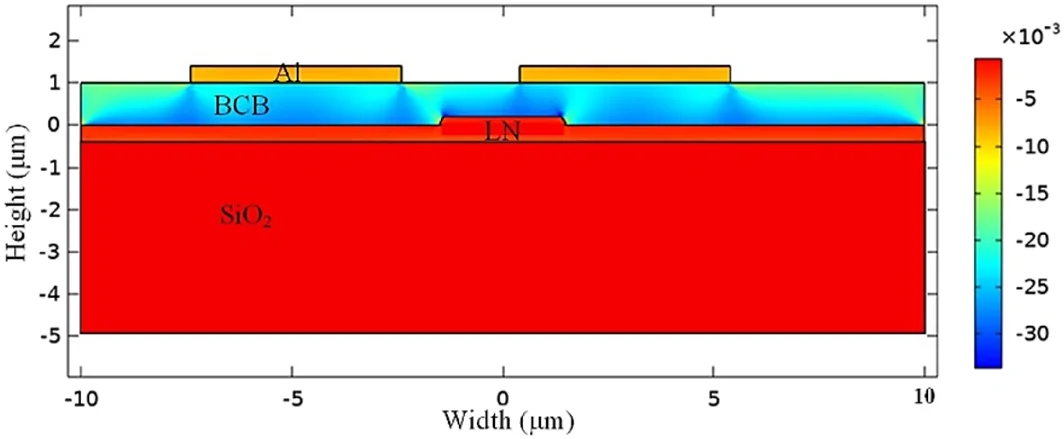
Calculated strain distribution in the BCB film.

This strain modulates the refractive index of BCB via the photo‐elastic effect. The refractive index change Δ*n* can be approximately expressed as follows [34]:

(2) $$\Delta n=-\frac{1}{2}n^{3}\cdot \Delta \varepsilon \cdot \left[p_{12}-\nu \left(p_{11}+p_{12}\right)\right],$$

where *p*_11_and *p*_12_ are the elasto‐optical coefficients of the BCB film, *ν* is Poisson's ratio of the BCB film. Using *p*_11_ ≈ 0.338, *p*_12_ ≈ 0.247, and *ν* ≈ 0.34 from literature [34], the calculated refractive index change of the BCB film is Δ*n* ≈ 0.0024. Because of the periodicity of the Al structure, the change in the refractive index of the BCB film is also periodic, thus forming an LPWG with the same period as the Al structure. A key advantage of this method is that it leverages the residual stress induced by the fabrication process, typically regarded as a detrimental factor, to achieve non‐invasive fabrication of efficient gratings, thereby laying the foundation for stable low‐loss device performance.

Based on the above calculated grating period and index modulation, the transmission spectrum of the designed LPWG was simulated using a grating analysis tool (GratingMOD, Rsoft). As shown in Figure 3a, the grating length *L* required for complete coupling is 135 periods when the lateral grating offset *d*_g_ is set to 1 μm. The coupling efficiency of the LPWG is determined by the refractive index modulation depth, the lateral grating offset *d*_g_, and the grating length *L*. A key practical challenge is the control of *d*_*g*_, which is susceptible to photolithographic alignment errors. Simulation results reveal that a deviation in *d*_g_ as small as 150 nm from its design value degrades the contrast at the resonant wavelength by approximately 18 dB, as shown in Figure 3b. In addition, a change in grating length by 30 periods results in a contrast reduction of approximately 19 dB at the resonant wavelength, as shown in Figure 3a. Therefore, fabricating a set of gratings with slightly different lengths can help compensate for coupling‐efficiency fluctuations induced by lateral offsets *d*_g_, thereby improving process tolerance and fabrication yield.

**FIGURE 3:**
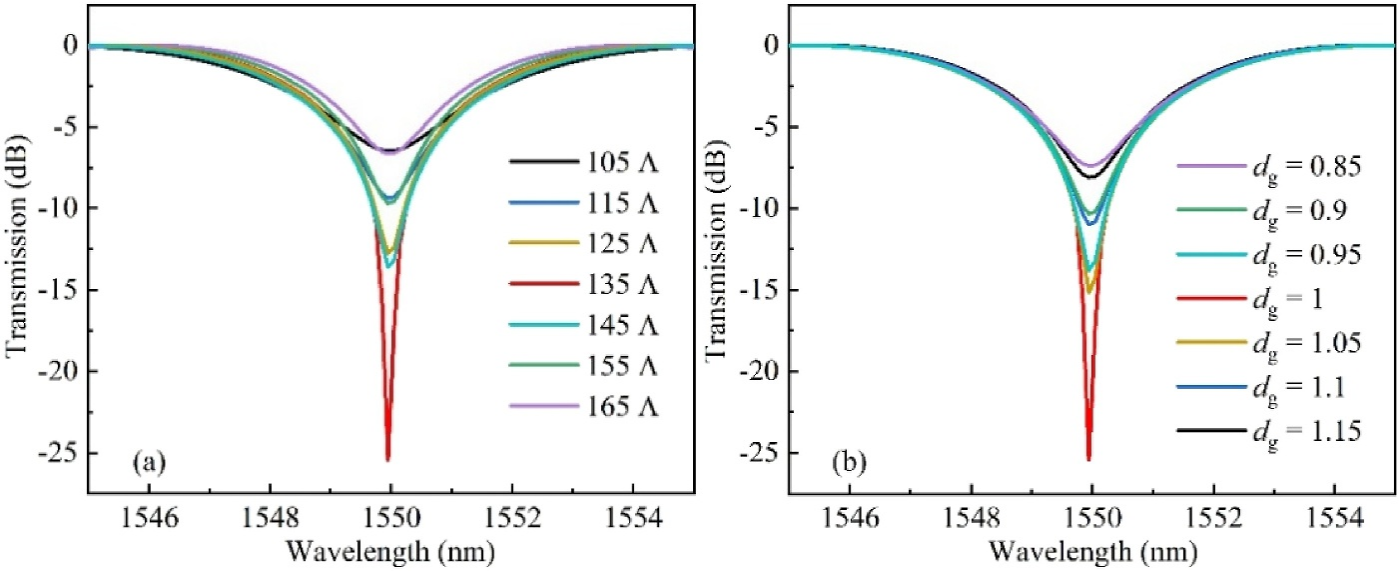
Calculated transmission spectra of the stress‐induced LPWG for (a) different grating lengths *L* (*d*_g_ = 1 μm) and (b) different lateral offsets *d*_g_ (*L* = 135 Λ).

It is worth noting that the proposed Al structures enable not only a stress‐induced grating in the BCB cladding but also an EO‐induced grating in the TFLN ridge waveguide when a voltage is applied across the modulation electrodes shown in Figure 1. Notably, the periods of the two gratings are nearly identical. Because the mode coupling introduced by the EO grating involves both the fundamental mode and the first‐order mode, precisely calculating their respective refractive index modulation is challenging. For simplicity, we evaluated the EO index modulation using the overlap integral between the optical field of the fundamental mode and the modulating electric field, and then investigated the transmission spectrum of the EO grating. Using the same device parameters (*w*_c_ = 2.8 μm, *h*_c_ = 280 nm, Λ = 39.13 μm, *L* = 135 Λ, *d*_g_ = 1 μm), we first employed COMSOL to calculate the optical field distribution of the *z*‐polarized fundamental mode and the static electric field distribution in the TFLN ridge waveguide under an applied voltage of 100 V, along with their overlap integral. Based on this calculation, the refractive index modulation is estimated to be approximately 1.33 × 10^−5^/V. We then investigated the transmission spectrum using GratingMOD, neglecting the stress‐induced index change in the BCB film and assuming a uniform EO index modulation in the TFLN across the grating region. Figure 4 shows the simulated transmission spectra for different applied voltages (from 23.5 to 32.5 V). Within this voltage range, the resonance contrast first increases with voltage, reaches 22.5 dB at 28 V (corresponding to a refractive index modulation of 3.7 × 10^−4^), and then decreases as the voltage moves away from 28 V.

**FIGURE 4:**
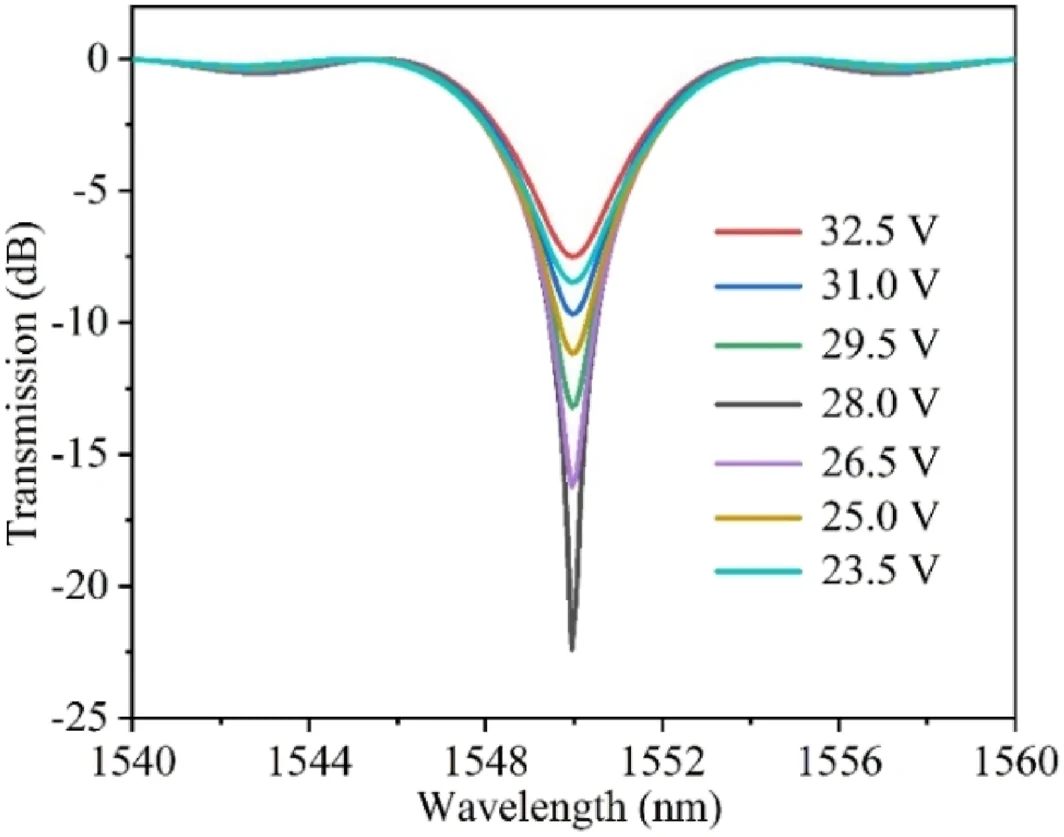
Simulated transmission spectra of the EO‐induced grating for different applied voltages from 23.5 to 32.5 V, with a resonance contrast of 22.5 dB at 28 V.

## 3 Fabrication and Charaterizations

Following the designed parameters as closely as possible, we fabricated the proposed stress‐induced LPWG using the standard photolithography and the dry etching processes on a commercial X‐cut TFLN wafer. This TFLN wafer consists of a 600‐nm‐thick single‐crystal LN film directly bonded to a 4.7‐μm‐thick silicon dioxide layer on a 500‐μm‐thick silicon substrate. First, a ∼160‐nm thick chromium (Cr) hard mask was deposited onto the TFLN wafer by radio‐frequency magnetron sputtering. The pattern of the straight channel waveguides was then defined by the standard photolithography and transferred into the Cr layer via chemical etching. Subsequently, the designed TFLN rib waveguides were formed by inductively coupled plasma (ICP) etching. The etching depth is 260 nm, which is very close to the design value. After the residual Cr film was removed with a dechroming solution, a 1‐μm thick BCB film was spin‐coated on the wafer as the top cladding. Finally, a periodic Al pattern was formed on the BCB layer through sequential steps of thermal evaporation, precision‐aligned photolithography, and lift‐off process. Special attention was paid to control the lateral offset *d*_g_ during the photolithography, ensuring that deviation from the design value remained within the process tolerance, which is critical for achieving a device with excellent performance.

After fabrication, the wafer was cleaved to form input and out facets. Considering the inevitable design and fabrication errors, as well as the high susceptibility of the resonant wavelength to the grating parameters, tens of LPWG were fabricated on the same chip. These LPWG arrays share an identical TFLN rib waveguide dimensions but slightly different grating period, length, and lateral offset, hereby maximizing the likelihood of achieving a device with excellent performance. A photograph of the fabricated chip comprising tens of LPWG is shown in Figure 5a. Furthermore, the microscopic image of the Al structure in Figure 5b confirms that a well‐defined periodic structure.

**FIGURE 5:**
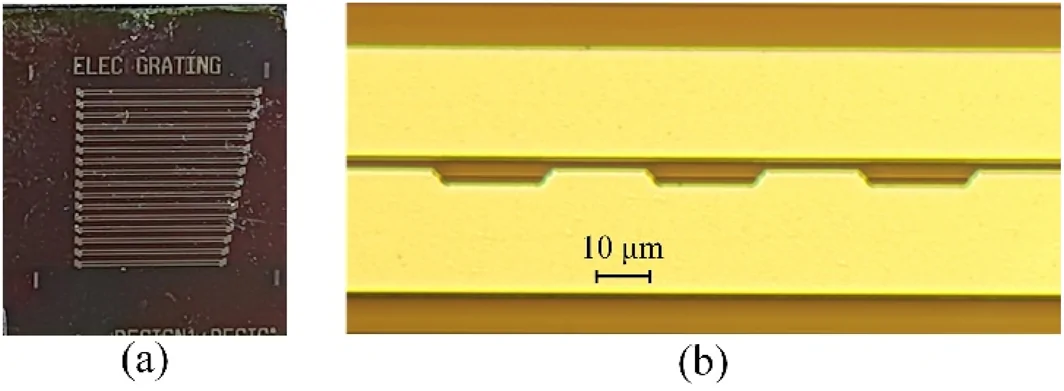
(a) Photograph of the fabricated chip showing tens of LPWG, (b) microscopic image of the Al structure.

To characterize the fabricated stress‐induced LPWG, light from an amplified spontaneous emission (ASE) source (B&A, AS4600, 1525–1605 nm) was launched into the device under test via a lensed single‐mode fiber (SMF). The polarization state was controlled using an in‐line fiber polarizer followed by a polarization controller. To ensure excitation of only the fundamental mode, the lensed SMF was carefully aligned to inject light at the center of the input waveguide facet. The output light from the LPWG was collected by another lensed SMF and measured using an optical spectrum analyzer (OSA; Anritsu MS97740A). The measurement was performed at a constant room temperature of 22°C. The transmission spectra of all LPWGs on the chip were measured sequentially and normalized to the fiber‐to‐fiber reference spectrum.

Figure 6 shows the normalized transmission spectra of the optimal device for the *z*‐ and *x*‐polarized input light. The key parameters (*w*_c_, *h*_c_, *d*_g_, Λ, *L*) of this grating are (2.9, 0.26, 1.2, 38.5, and 5005 μm). For *z*‐polarized input light, a resonant dip with a contrast of 19.65 dB and a 3‐dB bandwidth of approximately 5 nm is observed at 1542.68 nm, indicating a strong coupling achieved with the stress‐induced grating. For *x*‐polarized input light, due to the structural anisotropy and material birefringence, the resonant shifts to 1553.43 nm with a contrast of 12.38 dB. These results demonstrate that a functional grating can be realized by utilizing the residual stress, without the need for waveguide core etching.

**FIGURE 6:**
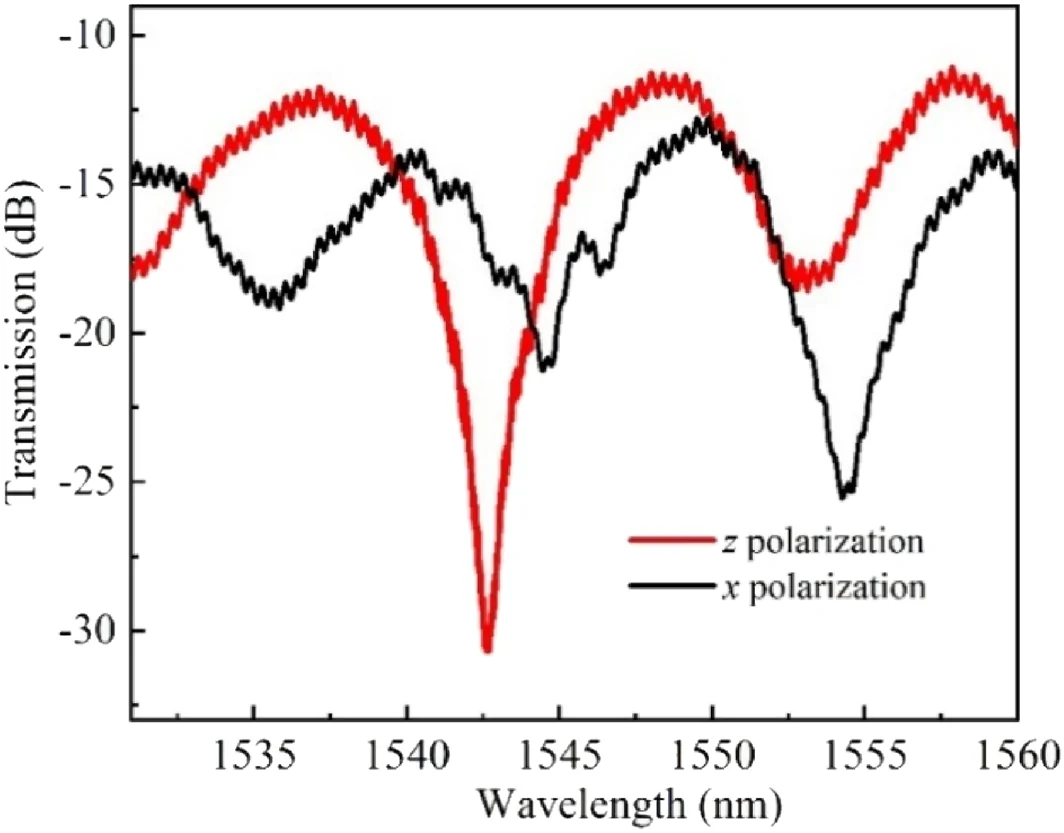
Normalized transmission spectra of the stress‐induced LPWG for the *z*‐ and *x*‐polarized input light.

### 3.1 EO Tuning Characteristics

The EO tuning characteristics of the optimal LPWG were investigated by applying positive and negative tuning voltages to the modulation electrodes. Here, a positive (negative) voltage refers to a positive (negative) potential difference between the periodic electrode and the straight electrode. The normalized transmission spectra for *z*‐polarized input light at different positive (0–40 V) and negative (0 to −40 V) tuning voltages are shown in Figure 7a,b, respectively, revealing a markedly distinct tuning behavior. As shown in Figure 7a, under positive voltage, the resonant wavelength remains essentially unchanged, whereas the contrast increases with the increase of the tuning voltage. Meanwhile, the insertion loss stays almost constant. In contrast, under negative voltage, as the voltage magnitude increases, the resonant wavelength exhibits a linear blueshift, the contrast decreases linearly, and the insertion loss increases gradually at a rate of ∼0.075 dB/V, as shown in Figure 7b. The possible reasons for this phenomenon include: First, due to the design and fabrication imperfections, the coupling coefficient of the stress‐induced grating and the grating length are not perfectly matched, resulting in an isolation of only about 19 dB, which leaves room for improvement. Second, the refractive index change induced by the EO effect depends on the direction of the electric field, that is, the polarity of the applied voltage. In this work, a periodic positive tuning voltage along the waveguide causes a periodic increase in the LN refractive index, thereby creating an LPWG in the LN core with the same period as the BCB cladding grating. The superposition of these two gratings enhances the coupling coefficient. As the tuning voltage magnitude increases, the coupling coefficient progressively matches better with the grating length, leading to a gradual improvement in the contrast. Finally, for a negative tuning voltage, the EO effect induces a periodic decrease in the refractive index of the LN along the waveguide direction, similarly generating an LPWG with a period matching that of the BCB cladding grating. The superposition of these two gratings weakens the overall grating strength. As the tuning voltage magnitude increases, the grating strength gradually diminishes, the period undergoes slight changes, and the coupling efficiency decreases, ultimately resulting in a blueshift of the resonant wavelength and a reduction in contrast. For the case of tuning with negative voltage, the linear fits reveal a resonant wavelength tuning efficiency of 25.1 pm/V and a resonant contrast variation of approximately −0.28 dB/V, respectively, as shown in Figure 7e.

**FIGURE 7:**
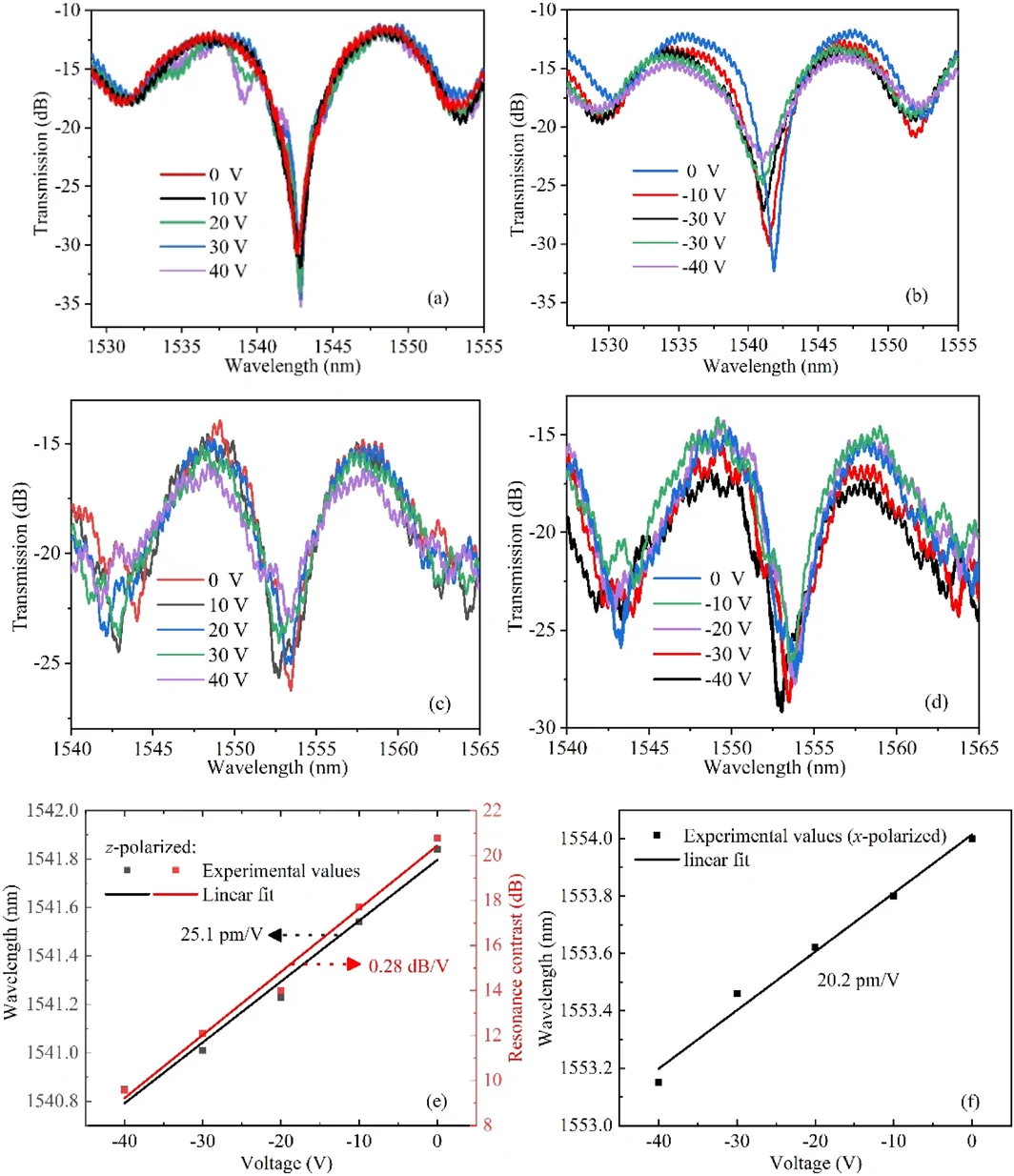
Normalized transmission spectra of the optimal LPWG under different tuning voltages for the (a), (b) *z*‐ and (c), (d) *x*‐polarized input light, (a) and (c) show results under positive voltage from 0 to +40 V, while (b) and (d) correspond to negative voltage from 0 to −40 V. (e) Resonant wavelength and contrast as functions of positive tuning voltage for the *z*‐polarized input, together with the linear fitting line. (f) Resonant wavelength versus negative tuning voltage for the *x*‐polarized input, together with the linear fitting line.

The normalized transmission spectra for the *x*‐polarized input light under different positive (0–40 V) and negative (0 to −40 V) tuning voltages are presented in Figure 7c,d, respectively. These results show a tuning trend similar to that observed under the *z*‐polarized input. As shown in Figure 7c, for the positive tuning voltages, the resonant wavelength remains nearly unchanged, while the resonant contrast decreases by approximately 3.1 dB in total. In contrast, for the negative tuning voltages, as shown in Figure 7d, the resonant wavelength blueshifts and the resonant contrast increases slightly by about 2 dB, corresponding to a wavelength tuning efficiency of 20.2 pm/V, as shown in Figure 7f. This behavior can be attributed to the fact that the EO coefficient for the *x*‐polarization (*γ*_13_) is significantly smaller than that for the *z*‐polarization (*γ*_33_). Consequently, the EO‐induced grating for the *x*‐polarization is much weaker than the stress‐induced grating, leading to low tuning efficiencies in both resonant wavelength and contrast, which align with the anisotropic EO properties of LN. Additionally, for both positive and negative tuning voltages, the insertion loss increases slightly with the magnitude of the tuning voltage, at a rate of ∼0.075 dB/V. Since we did not adjust the fiber‐waveguide alignment during our tuning measurements, it can be assumed that the coupling loss remained unchanged. Therefore, the increase in insertion loss should arise from an increase in transmission loss. We speculate that this increase is due to tuning‐induced changes in the mode field distribution, which slightly enhance either the sidewall scattering loss of the waveguide or the absorption loss induced by the metal electrodes.

To characterize the EO tuning response of the fabricated grating, we used a tunable laser and a signal generator to measure the output power variation of the grating at the resonance wavelength during the EO and TO tuning under a square‐wave electrical signal drive. The measured EO tuning response for the *z*‐polarized input light is shown in Figure 8, which indicates a rise time of 45.08 ns and a fall time of 95.15 ns, confirming a fast EO tuning response. The 3 dB bandwidth of the EO tuning is calculated to be about 7.8 MHz.

**FIGURE 8:**
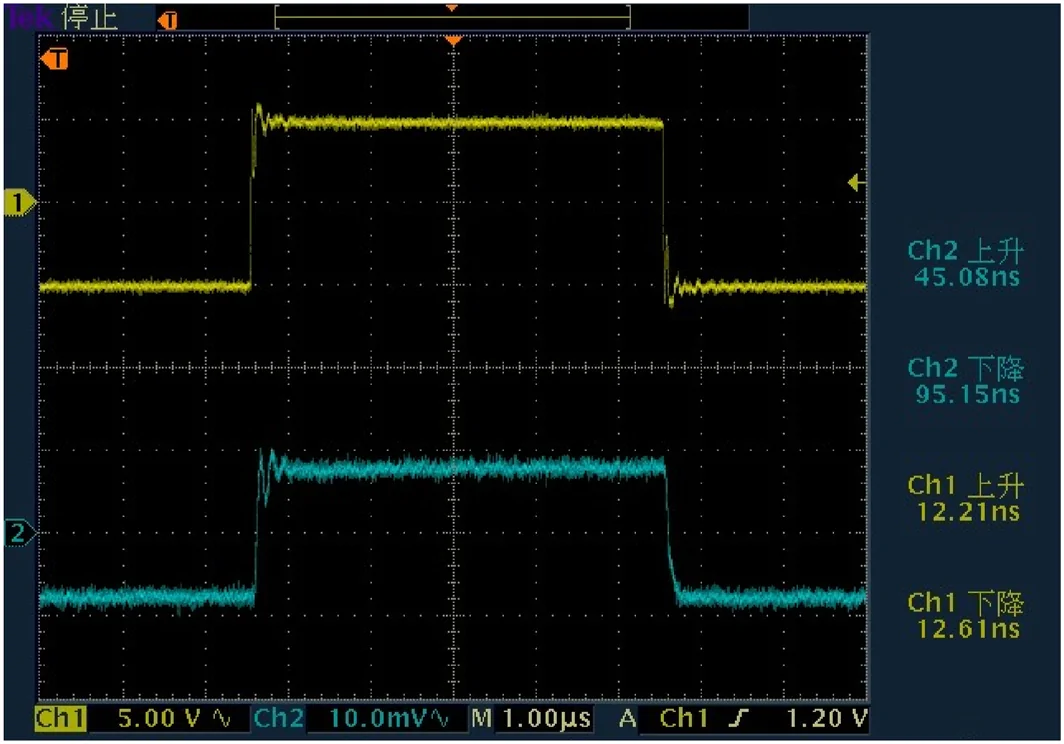
EO tuning response measured at the resonance wavelength of the grating.

### 3.2 TO Tuning Characteristics

As mentioned above, the periodic Al structure can also serve as a micro‐heater. By passing a current through it to generate Joule heat, we investigated the TO tuning characteristics of the optimal LPWG. Figure 9a presents the normalized transmission spectra for *z*‐polarized input light at different heating powers ranging from 0 to 128 mW. As shown in Figure 9a, with increasing heating power, the resonant wavelength exhibits a continuous blueshift, the contrast remains almost unchanged, and the insertion loss gradually increases at a rate of approximately 0.016 dB/mW. The linear fitting in Figure 9b yields a TO tuning efficiency of 11.4 pm/mW. Furthermore, Figure 9c shows the tuning characteristics for the *x*‐polarized input light over a tuning power range from 0 to 130 mW. The tuning behavior is similar to that observed for the *z*‐polarized input light described above; however, the insertion loss remains almost unchanged and the tuning efficiency is slightly lower, approximately 8.4 pm/mW, as shown in Figure 9d.

**FIGURE 9:**
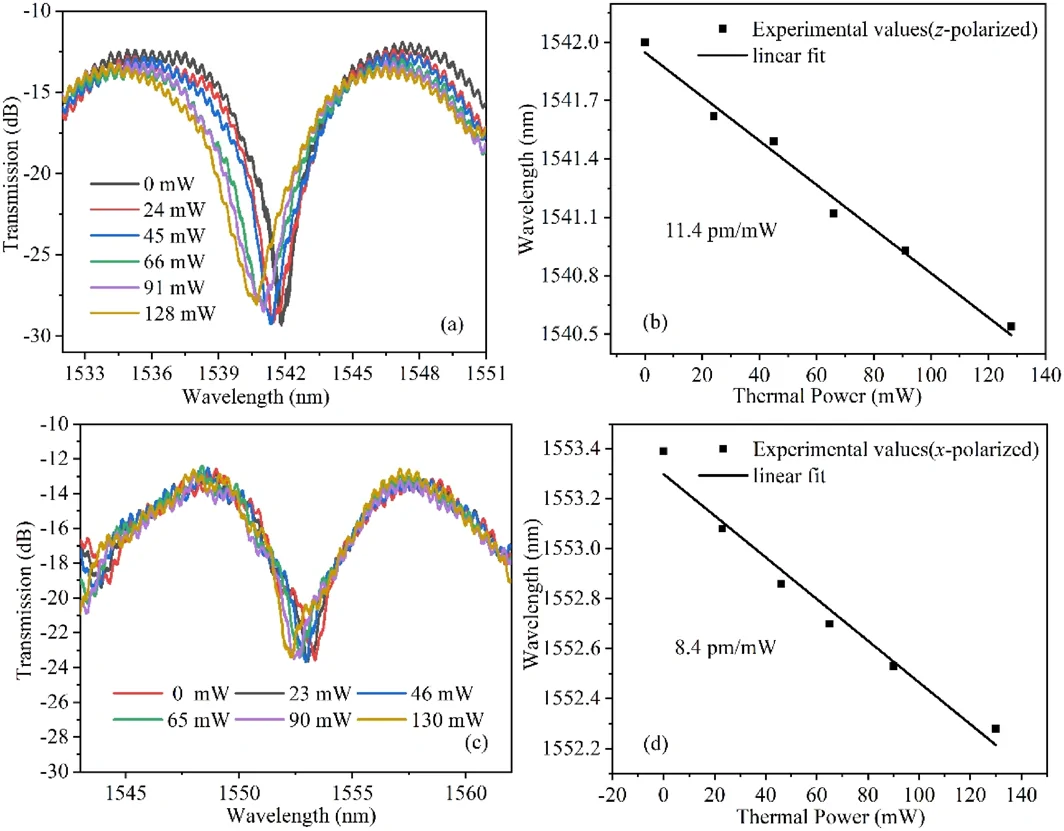
Normalized transmission spectra of the optimal LPWG under different heating powers for the (a) *z*‐ and (c) *x*‐polarized input light. Resonant wavelength as a function of heating power for the (b) *z*‐ and (d) *x*‐polarized input, together with the linear fitting line.

Unlike the EO tuning, the TO tuning here affects both the BCB cladding and the TFLN ridge waveguide core simultaneously. Because of the gradual distribution of the temperature field, the boundaries of the index modulation induced by the TO effect are less defined. Furthermore, considering the negative TO coefficient of BCB, when TO tuning is performed using the periodic micro‐heater, the thermally induced grating in the BCB cladding will partially counteract the stress‐induced grating. This occurs because the two gratings share a similar period but produce opposite refractive index modulation (stress increases the index, whereas the TO effect decreases it). As the heating power increases, the grating in the BCB cladding gradually weakens, whereas the thermal grating within the TFLN core gradually strengthens. Because LN has a positive and anisotropic TO coefficient, the overall effect resembles a transfer of the grating from the BCB cladding to the TFLN core. However, due to the anisotropic nature, the grating strengths for the two orthogonal polarizations differ slightly, resulting in different tuning efficiencies.

TO tuning response was also measured for the *z*‐polarized input light and the result is shown in Figure 10, indicating a rise time of 24.19 μs and a fall time of 27.11 μs.

**FIGURE 10:**
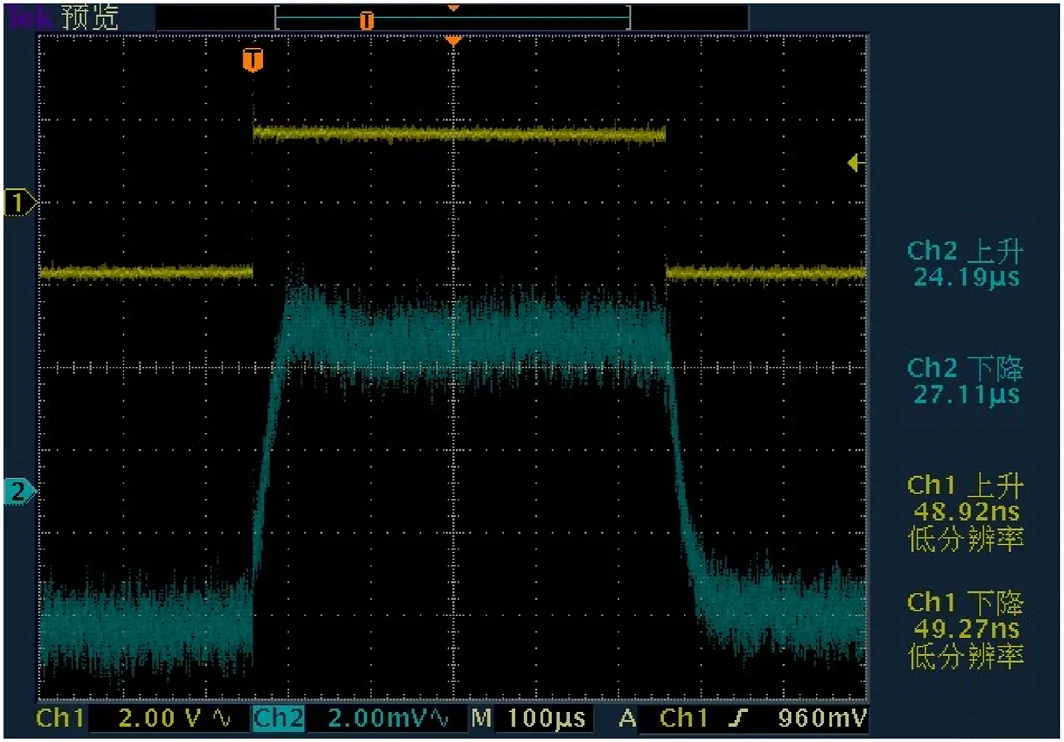
TO tuning response measured at the resonance wavelength of the grating.

It should be noted that for our proposed grating, the EO or TO tuning mechanism can operate independently with virtually no crosstalk. In our device, the EO electrodes (positive and negative) are separated by air, and no DC or low‐frequency current flows through the LN or BCB cladding during EO tuning. Consequently, no resistive Joule heating is generated, and the TO effect arising from Joule heating is fundamentally avoided by design. For thermal tuning, a DC voltage (≤ 2 V) is applied to the periodic electrode, with the positive and negative electrodes separated by ∼5 mm (equal to the grating length), producing a weak electric field parallel to the waveguide (light propagation direction). In this field direction, the EO coefficient *γ*_13_ of LN is also very small (= 8.9 pm/V@1550nm). Therefore, it does not induce any significant EO index change, and the EO contribution during thermal tuning is negligible.

### 3.3 Environmental Temperature Sensitivity

We further evaluated the stability of the device against environmental temperature fluctuations. The chip was placed on a metal platform equipped with a thermoelectric cooler (TEC) for temperature control. The normalized transmission spectra for the *z*‐ and *x*‐polarized input light, measured at ambient temperature ranging from 22°C to 32°C, are shown in Figure 11a,c, respectively. For both polarizations, the resonant wavelength exhibits a blueshift with increasing ambient temperature, while the contrast remains almost unchanged. The linear fittings shown in Figure 11b,d indicate temperature sensitivities of 0.175 and 0.154 nm/°C for the *z*‐ and *x*‐polarized input, respectively. This drift mainly originates from stress state changes due to material thermal expansion and the intrinsic TO effect of the materials.

**FIGURE 11:**
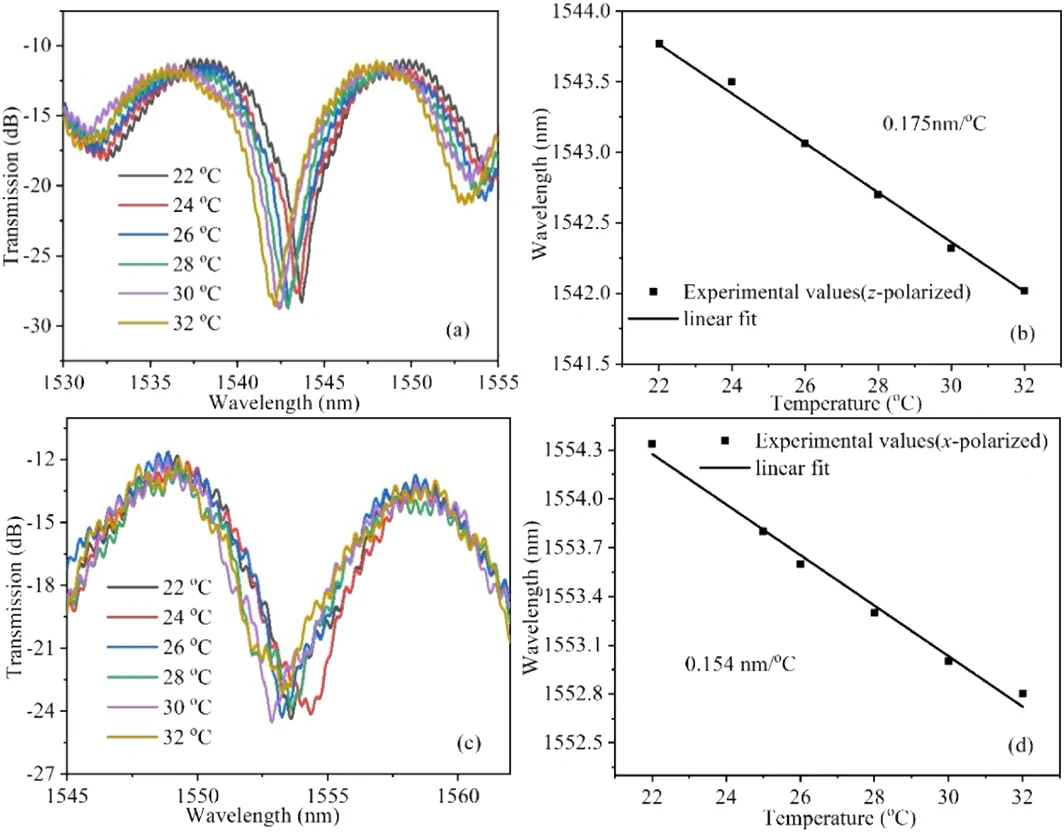
Normalized transmission spectra of the optimal LPWG under different ambient temperatures for the (a) *z*‐ and (c) *x*‐polarized input. Resonant wavelength as a function of the ambient temperature for the (b) *z*‐ and (d) *x*‐polarized input, together with the linear fitting line.

### 3.4 EO and TO Tuning Characteristics After Annealing

To gain deeper insight into the synergy and competition between the stress and EO effects, we performed an annealing treatment on the optimal LPWG at approximately 270°C. We then first investigated its EO tuning characteristics for the *z*‐polarized input light with tuning voltages ranging from −60 to +60 V, and the results are shown in Figure 12a. Compared to the unannealed device, the annealed grating shows notable improvements in tuning linearity and efficiency under both positive and negative tuning voltages. Unlike the unannealed case where only negative voltages shifted the resonant wavelength, both polarities became effective after annealing. The tuning efficiency of the resonant wavelength increased from 25.1 (before annealing) to 28.5 pm/V (after annealing), whereas the tuning efficiency of the contrast changed from −0.28 (before annealing) to −0.1 dB/V (after annealing), as shown in Figures 7e and 12b. This is because annealing effectively releases the residual stress between the Al structures and the BCB cladding [35], weakening the stress‐induced grating (i.e., reducing the initial index modulation). This allows the EO‐induced index modulation to become dominant, yielding a more linear and symmetric tuning response due to the intrinsic linearity of the Pockels effect. Consequently, annealing, which partially erases the stress‐induced index modulation, reduced the static (at 0 V) resonant contrast to about 11.2 dB while also enabling a dramatically expanded tuning range, allowing it to be enhanced to 31.86 dB under +60 V or completely suppressed under −60 V.

**FIGURE 12:**
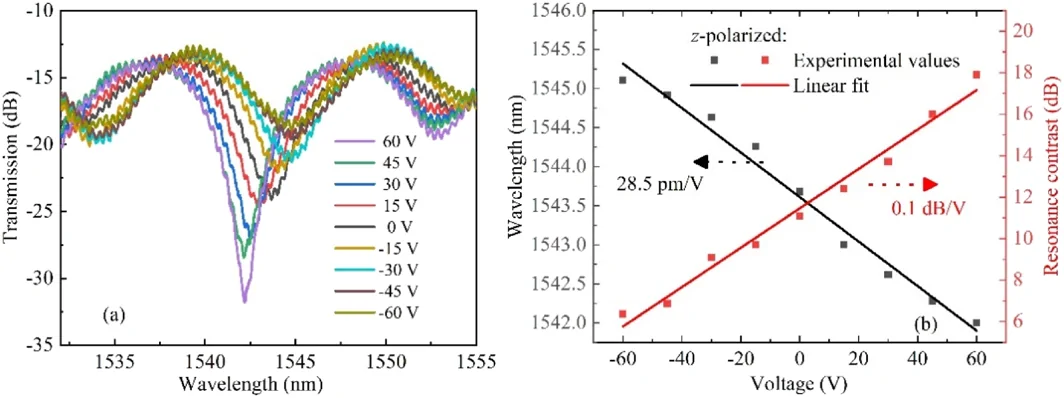
(a) Normalized transmission spectra for the *z*‐polarized input light of the optimal LPWG after annealing at 270°C, measured at applied negative voltages from +60 to −60 V. (b) Resonant wavelength and contrast versus the tuning voltage, together with the linear fitting lines.

Subsequently, we investigated its TO tuning characteristics for the *z*‐polarized input light with heating power ranging from 0 to 96 mW, and the results are shown in Figure 13a. After annealing, the resonant wavelength still blueshifts as the heating power increases, meanwhile, the tuning efficiency of the resonant wavelength notably rises from 11.4 (before annealing) to 18.6 pm/mW (after annealing), as shown in Figures 9b and 13b. This enhancement can be attributed to the effective weakening of initial index modulation of the stress‐induced grating by annealing. Therefore, based on the prior analysis of the TO tuning, it can be inferred that a stronger thermally induced grating forms in both the BCB cladding and the TFLN core under the same applied heating power as in the unannealed case, resulting in increased thermal tuning efficiency. In summary, the annealing treatment significantly improve both EO and TO tuning performance, including linearity and efficiency.

**FIGURE 13:**
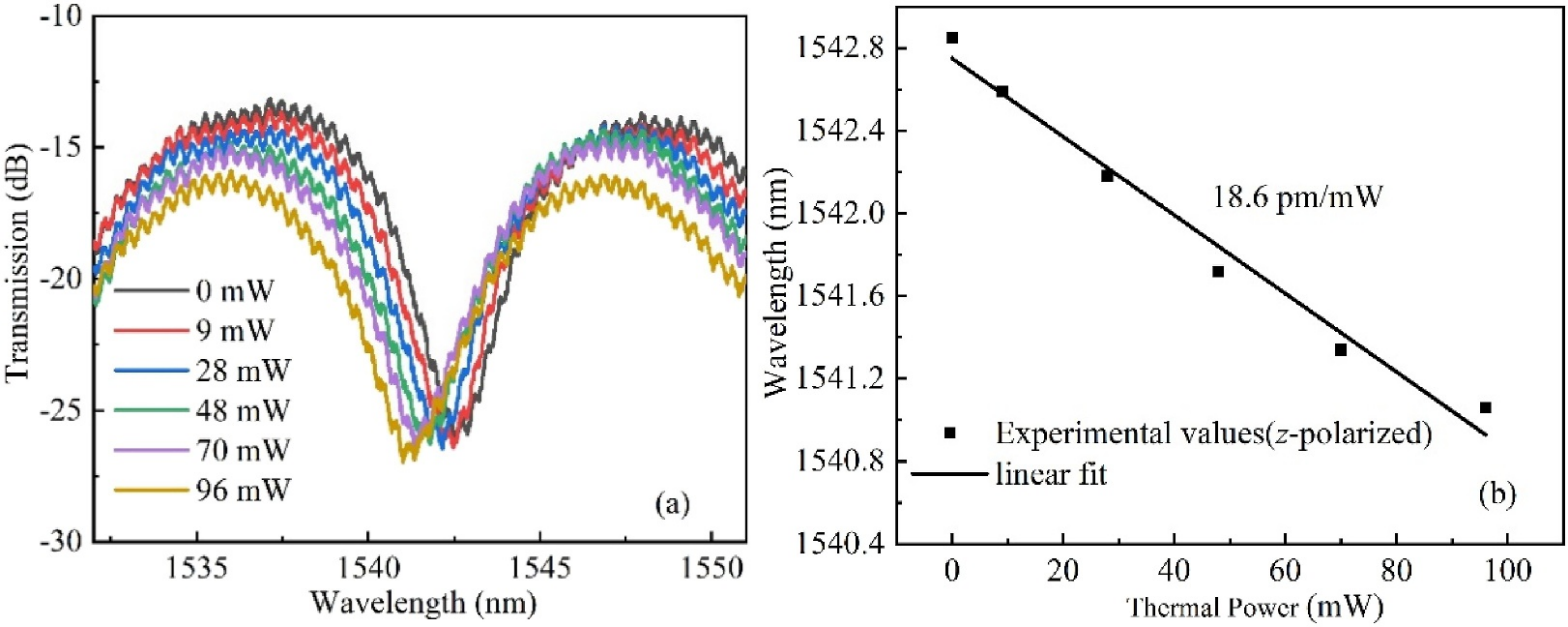
(a) Normalized transmission spectra for the *z*‐polarized input light of the optimal LPWG after annealing at 270°C, measured at applied heating power ranging from 0 to 96 mW. (b) Resonant wavelength and contrast versus the heating power, together with the linear fitting lines.

## 4 Conclusion

We have demonstrated a dynamically tunable LPWG on a hybrid polymer and TFLN platform. The grating is formed based on the stress‐induced index modulation and can be tuned using both EO and TO tuning mechanisms. The optimal device we fabricated exhibits static resonant contrasts of 12.38 dB at 1553.43 nm for the *x*‐polarized input light and 19.65 dB at 1542.68 nm for *z*‐polarized input light. For the unannealed device, dynamic EO tuning for the resonant wavelength can be achieved with efficiencies of 20.2 and 25.1 pm/V for the *x*‐ and *z*‐polarized input light, respectively. Meanwhile, for the *z*‐polarized input light, the resonant contrast can also be modulated at −0.28 dB/V under negative tuning voltage. The integrated micro‐heater provides a TO tuning efficiency of 11.4 pm/mW. Most importantly, we found that the annealing treatment significantly improves both EO and TO tuning performance, including linearity and efficiency. Annealing reduces the initial strength of the stress‐induced grating, which in turn increases the EO tuning efficiency to 28.5 pm/V and the TO tuning efficiency to 18.6 pm/mW. Moreover, the tuning range of the resonant contrast is also expanded, allowing the contrast to be enhanced from 24.18 (at 0 V) to 31.86 dB under +60 V, or to be completely suppressed under −60 V. The excellent tuning ability in both resonant wavelength and contrast makes the proposed LPWG a versatile component for applications in optical communications and information processing.

## Author Contributions

**Jiayao Deng:** data curation, investigation, validation, writing – original draft, formal analysis, conceptualization. **Hao Yao:** methodology, validation. **Yuzhe Sun:** investigation, data curation. **Hao Zhang:** validation. **Yinglu Zhang:** methodology. **Jieyun Wu:** data curation, formal analysis. **Kaixin Chen:** writing – review and editing, supervision, project administration, funding acquisition, conceptualization.

## Conflicts of Interest

The authors declare no conflicts of interest.

## Supporting information


Supporting Information S1

## Data Availability

The data that support the findings of this study are available from the corresponding author upon reasonable request.
