# Supplementary material for: A Long‐Period Grating Formed by Residual Stress on Thin‐Film Lithium Niobate With Efficient Electro‐Optic and Thermo‐Optic Tuning
Source: Nanophotonics. 2026 May 5;15(9):e70118. doi: 10.1002/nap2.70118 (PMC13642066; doi:10.1002/nap2.70118)
Supplement: Supplementary file 1 [file NAP2-15-e70118-s001.docx]

Supporting Information

A Long-Period Grating Formed by Residual Stress on Thin-Film Lithium Niobate with Efficient Electro-Optic and Thermo-Optic Tuning

Jiayao Deng, Hao Yao, Yuzhe Sun, Hao Zhang, Yinglu Zhang, Jieyun Wu, and Kaixin Chen*

School of Optoelectronic Science and Engineering, University of Electronic Science and Technology of China, Chengdu, China

**Corresponding Author: chenkx@uestc.edu.cn*

**Outline**

1. **Theoretical derivation of stress-induced refractive index changes in the BCB film**
2. **The fabrication process of the device.**

**References**

1. **Theoretical derivation of stress-induced refractive index changes in the BCB film**

For an isotropic medium, the change in the optical refractive index tensor with respect to the strain tensor is governed by the photoelastic effect, generally expressed as:

$\text{Δ}\left(\frac{1}{n^{2}}\right)_{ij}=\sum_{j} p_{ij}\cdot\varepsilon_{j}$ (S1)

where *n* is the refractive index of the medium, *p*_ij_ is the strain-optic coefficient tensor of the medium, and *ε*_j_ are the strain components. In the principal coordinate system (*x*, *y*, *z*) of an isotropic medium, this relationship simplifies to the refractive index changes along the three principal axes:

$\text{Δ}n_{x}=-\frac{1}{2}n_{cl}^{3}\left(p_{11}\varepsilon_{x}+p_{12}\varepsilon_{y}+p_{12}\varepsilon_{z}\right)$ (S2)

$\text{Δ}n_{y}=-\frac{1}{2}n_{cl}^{3}\left(p_{11}\varepsilon_{y}+p_{12}\varepsilon_{x}+p_{12}\varepsilon_{z}\right)$ (S3)

$\text{Δ}n_{z}=-\frac{1}{2}n_{cl}^{3}\left(p_{11}\varepsilon_{z}+p_{12}\varepsilon_{x}+p_{12}\varepsilon_{y}\right)$ (S4)

Here, *n*_cl_ = 1.538 is the stress-free refractive index of BCB at 1550 nm wavelength (measured in our laboratory). In the device presented in this work, a significant mismatch in thermal expansion coefficient exists between the periodic Al electrodes and the BCB cladding. During cooling after Al deposition, this mismatch induces a residual tensile stress in the BCB, primarily along the *x*-direction (perpendicular to the chip surface). Therefore, we assume that the principal stress state satisfies: *σ_x_* $\neq$ 0, *σ_y_* $\approx$ 0, *σ_z_* $\approx$ 0. According to the generalized Hooke's law, the corresponding strain components are:

$\varepsilon_{x}=\text{Δ}\varepsilon, \varepsilon_{y}=\varepsilon_{z}=-\nu\text{Δ}\varepsilon$ (S5)

where Δ*ε* is tensile strain of BCB, and *ν* = 0.34 is the Poisson's ratio of BCB. For the *z*-polarized light (with its electric field along the *z*-direction), which is the focus of this study, substituting the strain relations from Eq. (S5) into the refractive index change formula Eq. (S4) yields:

$\text{Δ}n_{z}=-\frac{1}{2}n_{\text{cl}}^{3}\cdot\text{Δ}\varepsilon\cdot[p_{12}-\nu(p_{11}+p_{12})]$ (S6)

The strain-optic coefficients *p*_11_ and *p*_12_ in Eq. (S6) must be converted from the experimentally measured stress-optic coefficients (*C*_1_ = -107$\times$10^-12^ m^2^/N and *C*_2_ = -30$\times$10^-12^ m^2^/N). This work uses the data for BCB thin films measured using the prism coupling technique from Ref. [1]:

$\begin{matrix} C_{1}=-\frac{n_{\text{cl}}^{3}}{2E}(p_{11}-2\nu p_{12}) \\ C_{2}=-\frac{n_{\text{cl}}^{3}}{2E}(p_{12}-\nu p_{11}-\nu p_{12}) \end{matrix}$ (S7)

Here, *E* = 2.9 GPa is the Young's modulus of BCB. Based on the given parameters, the strain-optic coefficients of BCB are calculated as *p*_11_≈0.338 and *p*_12_≈0.247.

To estimate the strain induced in the BCB film, we performed a thermal‑mechanical simulation using COMSOL Multiphysics. The strain originates from the significant mismatch between the thermal expansion coefficients of the patterned Al and the underlying BCB. Assuming a temperature drop of approximately 300 °C during the cooling phase after Al deposition, the simulation yields an average tensile strain Δ*ε* ≈ 2.6×10^−2^ in the BCB cladding region adjacent to the TFLN ridge waveguide.

Substituting the obtained strain Δ*ε*, the strain‑optic coefficients *p*_11_ and *p*_12_, and the other material parameters into Eq. (S6), the refractive‑index change induced by the thermal stress in the BCB cladding was calculated to be Δ*n* ≈ 0.0024. Such a magnitude of index modulation is fully sufficient to form an efficient long‑period grating.

2. **The fabrication process of the device.**

Figure S1 presents the fabrication process of the proposed device. The device is fabricated on an X-cut thin-film lithium niobate (TFLN) wafer (shown in Figure S1(a)), which consists of a 600-nm-thick single-crystal LN film directly bonded to a 4.7-μm-thick silicon dioxide layer on a 500-μm-thick silicon substrate. The fabrication process is detailed below:

First, a ~160‑nm‑thick chromium (Cr) hard mask was deposited onto the TFLN wafer by radio‑frequency magnetron sputtering. Standard i‑line photolithography was then used to define the straight waveguide pattern, which was transferred into the Cr layer by chemical etching. Subsequently, the pattern was transferred into the TFLN layer by inductively coupled plasma (ICP) etching, achieving an etch depth of 260 nm (see Figs. S1(b)–(f)). The residual Cr mask was removed with a dechroming solution. Figs. S2(a) and S2(b) show top‑view and cross‑sectional scanning electron microscopy (SEM) images of a fabricated straight waveguide, respectively. The sidewall angle is approximately 60° (Fig. S2(b)). A 1‑μm‑thick benzocyclobutene (BCB) film was spin‑coated onto the wafer as the upper cladding (Fig. S1(g)).Finally, a periodic Al pattern was formed on the BCB layer through sequential steps of thermal evaporation, precision‑aligned photolithography, and lift‑off. (Fig. S1(j)).

**Figure S1.** The fabrication process flow of the proposed device.

**Figure S2.** SEM images of the fabricated straight waveguide, (a) top view and (b) cross-sectional view.

[1].	F. Ay, A. Kocabas, C. Kocabas, A. Aydinli, and S. Agan, "Prism coupling technique investigation of elasto-optical properties of thin polymer films", *J. Appl. Phys.*, vol. 96, no. 12, pp. 7147–7153, 2004.
